# Atomic Layer Deposited TiO_2_ and Al_2_O_3_ Thin Films as Coatings for Aluminum Food Packaging Application

**DOI:** 10.3390/ma12040682

**Published:** 2019-02-25

**Authors:** Vanessa Dias, Homero Maciel, Mariana Fraga, Anderson O. Lobo, Rodrigo Pessoa, Fernanda R. Marciano

**Affiliations:** 1 Centro de Ciência e Tecnologia de Plasmas e Materiais (PlasMat), Instituto Tecnológico de Aeronáutica (ITA), São José dos Campos, SP 12228-900, Brazil; van_ametista@yahoo.com.br (V.D.); homero@ita.br (H.M.); 2 Laboratório de Nanotecnologia Biomédica, Universidade do Vale do Paraíba (Univap), São José dos Campos, SP 12244-000, Brazil; 3 Instituto Científico e Tecnológico, Universidade Brasil, São Paulo, SP 08230-030, Brazil; 4 Instituto de Ciência e Tecnologia, Universidade Federal de São Paulo (Unifesp), São José dos Campos, SP 12231-280, Brazil; mafraga@ieee.org; 5 LIMAV-Laboratório Interdisciplinar de Materiais Avançados, Universidade Federal do Piauí (UFPI), Teresina, PI 64049-550, Brazil; aolobo@pq.cnpq.br

**Keywords:** corrosion barrier, titanium dioxide, aluminum oxide, atomic layer deposition, linear sweep voltammetry, electrochemical impedance spectroscopy

## Abstract

Titanium dioxide (TiO_2_) and aluminum oxide (Al_2_O_3_) coatings have been investigated in a wide range of bio-applications due to their biodegradation and biocompatibility properties, that are key parameters for their use in the food packaging and biomedical devices fields. The present study evaluates and compares the electrochemical behavior of the non-coated, commercial resin-coated, TiO_2_-coated and Al_2_O_3_-coated aluminum in commercial beer electrolyte. For this, TiO_2_ and Al_2_O_3_ thin films were deposited on aluminum (Al) substrates using atomic layer deposition (ALD). The evaluation of the corrosion barrier layer properties was performed by linear sweep voltammetry (LSV) during 10 min and electrochemical impedance spectroscopy (EIS). In addition, profilometry, grazing incidence X-ray diffractometry (GIXRD), scanning electron microscopy (SEM) and Fourier-transform infrared spectroscopy (FT-IR) analyses were performed to investigate the physical and chemical properties of the pristine and / or corroded samples. TiO_2_ and Al_2_O_3_ films presented an amorphous structure, a morphology that follows Al substrate surface, and a thickness of around 100 nm. Analysis of LSV data showed that ALD coatings promoted a considerable increase in corrosion barrier efficiency being 86.3% for TiO_2_-coated Al and 80% for Al_2_O_3_-coated Al in comparison with 7.1% of commercial resin-coated Al. This is mainly due to the lower electrochemical porosity, 11.4% for TiO_2_-coated Al and 20.4% for Al_2_O_3_-coated Al in comparison with 96% of the resin-coated Al, i.e. an increase of up to twofold in the protection of Al when coated with TiO_2_ compared to Al_2_O_3_. The EIS results allow us to complement the discussions about the reduced corrosion barrier efficiency of the Al_2_O_3_ film for beer electrolyte once SEM and FT-IR analyzes did not show drastic changes in both investigated ALD films after the corrosion assays. The above results indicate that ALD TiO_2_ and Al_2_O_3_ films may be a viable alternative to replace the synthetic resin coatings frequently used in aluminum cans of use in the food industry.

## 1. Introduction

The food industry is always looking for new technologies for improving packaging techniques in order to maintain the food quality and assuring food safety. At the same time, many studies have been devoted to for the use of nanotechnology in food science because of the growing need by healthier and low-cost products. 

Aluminum (Al) is widely used in the food packaging industry because it is lightweight, recyclable, protects the stored liquid from various external factors such as moisture, microbiological contamination, among others [1]. However, some internal factors, such as the chemical processes promoted by the stored liquid, might result in degradation of Al surface resulting in corrosion and migration of metals [2,3,4].

The CO_2_-containing beverages such as beer and carbonated drinks have acidity and chlorides in the composition and, when packed in Al cans, can promote the corrosion process by coming into contact with the Al material. To mitigate this problem, commercial Al cans are internally spray coated by synthetic resins, generally natural or synthetic epoxy, that aims to protect the Al surface from direct contact with the beverage [4,5,6]. However, when the resin is not applied correctly or when the Al can undergoes mechanical impact or elevated temperatures, the occurrence of localized corrosion is verified due to the presence of pinholes, cracks or grains boundaries in the coatings [7]. Moreover, some resins contain bisphenol A (BPA), which is introduced into the coating during production. There is a concern that BPA may leach into the liquid contained inside beverage cans [8]. These problems may cause loss of product quality and integrity. In addition, if the contaminated product reaches the final consumer it can be harmful to human health [8,9].

Currently, need for rapid development of new internal coatings on Al cans has arisen in the food and beverage industry, mainly as a response to proposed volatile organic compound regulations and a desire to contain new and more aggressive food chemistries [10,11]. Acidity of commercial soft drinks varies greatly; the pH of beverages is typically between 2.3 to 4.0. For instance, Coke^TM^ is pH 2.38 and a beer is pH around 4.0 [12]. Thus, a coating should behave as a barrier throughout the range of pH solutions, avoiding reaching the underlying surface of the aluminum. 

Ceramic thin films have attracted much attention as protective barrier coatings due to their resistance to heat, corrosion, and wear [13,14,15]. Al_2_O_3_ and TiO_2_ thin films have been the focus of extensive research in recent years [16,17]. Al_2_O_3_ is the most readily studied thin film for corrosion protection, because it presents low porosity that prevents the solution from accessing the metal [18]. TiO_2_ is other important ceramic material used as corrosion protection layer of metals [19,20]. However, TiO_2_ has issues during film nucleation leading to high porosity [21]. The grain boundaries present in crystalline TiO_2_ are susceptible to species diffusion, which promote surface corrosion [22]. A possible solution is to deposit amorphous TiO_2_ thin films.

There are many well-studied methods for applying corrosion protective coatings including reactive sputtering, spray pyrolysis, electrochemical deposition, and chemical vapor deposition (CVD). Nevertheless, such preparation methods usually leave cracks or pinholes in the coatings [23]. Recently, several groups have explored atomic layer deposition (ALD) technique as a mean to form various metal oxide corrosion protection layers such as Al_2_O_3_, TiO_2_, ZnO, HfO_2_, etc., on various types of surfaces. ALD enables to obtain pinhole-free thin films with precise control over composition and thickness [20]. Furthermore, the ALD stands out in relation to CVD and PVD technologies, because it allows to deposit more conformal layers even on complex 3D shaped substrates [24]. 

The use of ALD thin films for the corrosion protection of metallic substrates was speculated for the first time in the 1990s by Matero et al., which demonstrated that ALD TiO_2_/Al_2_O_3_ films deposited on stainless steel have good corrosion resistance [22]. Since then, much research has been done to investigate the limits of application of this technique. Shan et al. investigated the improvement of corrosion resistance of CrN coated stainless steel with ALD TiO_2_ film [20,23]. Du et al. reported on the chemical corrosion protection of aluminum mirrors by ALD SiO_2_ coatings [25]. Marin et al. evaluated the long-term corrosion resistance performances of three different ALD single layer strategies (TiO_2_, Al_2_O_3_ and AlN) applied on AISI 316 substrates [24]. Recently, Daubert et al. investigated the corrosion protection of the copper using ALD Al_2_O_3_, TiO_2_, ZnO, HfO_2_, and ZrO_2_ thin films [18].

In food packaging, the application of ALD technique has already been discussed for the protection and increase of shelf life of products such as paperboard with Al_2_O_3_ and TiO_2_ coatings [26,27]. However, the synthesis of protective thin films using the ALD process in the field of aluminum cans for carbonated beverages has not been reported. 

In this article, due to the lack of studies on the use of ALD Al_2_O_3_ and TiO_2_ films as corrosion protection of Al in low pH environment, we investigated the feasibility of these coatings for such application showing their preparation and initial corrosion resistance (10 min) when deposited on aluminum substrates. Profilometry, grazing incidence X-ray diffractometry (GIXRD), scanning electron microscopy (SEM) and Fourier-transform infrared spectroscopy (FT-IR) analyses were performed to investigate the physical and chemical properties of the pristine and / or corroded samples. The evaluation of the corrosion resistance of the protected samples was performed by linear sweep voltammetry (LSV). Moreover, electrochemistry impedance spectroscopy (EIS) measurements were performed to investigate the electrical characteristics of the electrode–solution interface.

## 2. Materials and Methods 

### 2.1. Substrate Preparation

Commercial bare and resin-coated aluminum (AA3104 alloy, Latapack-Ball, Jacareí, Brazil) cans were cut into pieces of 1 × 1 cm^2^ and separated in four groups: (i) control or bare Al, (ii) resin-coated, (iii) TiO_2_-coated Al, and (iv) Al_2_O_3_-coated Al. For ALD coating, all samples were cleaned with distilled water and isopropyl alcohol, and then dried with N_2_ gas before being placed in the ALD reactor. The TiO_2_ and Al_2_O_3_ films were prepared by a Beneq TFS-200 equipment (Beneq Oy, Espoo, Finland), operating in thermal mode, at 100 °C and in the condition of 1000 reaction cycles. The choice of the process temperature is related to the good anticorrosive results of ALD metal oxides films reported by Sammelselg et al. [28]. Titanium tetrachloride (TiCl_4_) and trimethylaluminum (TMA) were used as metallic precursors and deionized water (H_2_O) as oxidant. Nitrogen (N_2_) of 99.999 % purity was used as purge gas. The corresponding ALD cycle time parameters for TiO_2_ films were 0.25, 2, 0.25 and 2 s for TiCl_4_ pulse, purge, H_2_O pulse and purge. While for Al_2_O_3_ films the cycle times were 0.15, 0.75, 0.15 and 0.75 s for the TMA pulse, purge, H_2_O pulse and another purge, respectively. These are optimal cycle time conditions investigated in early studies [29,30,31,32,33,34]. The vapors of TiCl_4_, TMA and H_2_O were led into the reaction chamber from external reservoirs kept with liquid TiCl_4_ (99.95 %, Sigma-Aldrich, São Paulo, Brazil), liquid TMA (97%, Sigma-Aldrich) and deionized water at temperature of 21 °C. A capillary tube, adapted to the reactor, was used to control the precursor flow injected into the ALD chamber, by action of the precursor vapor pressure only, i.e., no bubbling system was used. The base pressure of the reactor was lower than 10^−2^ mbar and during the deposition the gas pressure was maintained around 1.0 mbar through the insertion of 300 sccm of N_2_.

### 2.2. Film Characterization

The thickness of the as-deposited films was measured using a KLA Tencor P-7 profilometer (KLA Corporation, Milpitas, CA, USA). To characterize the structure of the as-deposited films, grazing incidence X-ray diffraction (GIXRD) method was used. GIXRD patterns were obtained at room temperature in a Shimadzu XRD 6000 goniometer (Shimadzu Corporation, Kyoto, Japan) using a copper target (CuKα radiation 1.5418 Å), 2θ from 20°–80°, at a scanning speed of 0.02° s^−1^, a voltage of 40 kV, and a current of 30 mA. To investigate the chemical bonds of the pristine and corroded samples, infrared measurements were performed on an ATR-FTIR PerkinElmer 400 IR spectrometer (PerkinElmer Brasil, São Paulo, Brazil) at a resolution of 2 cm^−1^. Each ATR spectrum was recorded with the blank ATR cell (PerkinElmer Brasil, São Paulo, Brazil) as the background. In addition, some analyzes of the surface morphology of the pristine and corroded samples were made with a field emission scanning electron microscope (FE-SEM) Tescan Mira 3 FEG (TESCAN Brno, s.r.o., Kohoutovice, Czech Republic) operated at 5 kV.

### 2.3. Electrochemical Measurements

All electrochemical measurements were conducted on Autolab 302N potentiostat/galvonostat (Metrohn Autolab B.V., Utrecht, the Netherlands) controlled by Nova 2.0 software (Metrohn Autolab B.V., Utrecht, the Netherlands). A standard three-electrode electrochemical cell (Metrohn Autolab B.V., Utrecht, the Netherlands) was used in electrochemical experiments. In the setup, saturated Ag/AgCl (3M KCl) (Metrohn Autolab B.V., Utrecht, the Netherlands) was used as a reference electrode, pure platinum coiled wire (Metrohn Autolab B.V., Utrecht, the Netherlands) as a counter electrode and the samples (i-iv) as working electrode. The exposed surface area of the tested samples was 0.78 cm^2^ and their back side have been sanded to improve the ohmic contact. For each experiment, the electrochemical cell was filled with 250 mL commercial bottled lager beer (Brasil Kirin, Itu, Brazil) (pH = 4.12 ± 0.02). All electrochemical assays were performed after 1 h immersion of pristine and coated Al samples in beer at the open circuit potential (OCP). The impedance spectra were obtained over the 100 mHz to 100 kHz frequency range with sine wave potential of 30 mV. The experiments were conducted at room temperature and a Kramers–Kronig routine was performed to ensure the linearity, causality and stability of the system through the measurements [35]. The EIS data was then modeled using equivalent circuit models, and curve fitting was performed using the Nova 2.0 software package. The fitted parameters have an uncertainty of ±15%.

The LSV tests were performed in voltage range of −0.65 to −0.35 V at scan rate of 1 mV s^−1^. The corrosion current (*i_corr_*) and corrosion potential (*V_corr_*) were obtained from LSV and Tafel plots; *i_corr_* was the current obtained from intercept of tangent lines on cathodic and anodic branches of Tafel plots at *V_corr_* [36]. The potential corresponding to zero current (current transition from cathodic to anodic) was assigned as *V_corr_*. These three parameters were easily obtained through signal analysis option of Nova software.

From these LSV parameters is possible to calculate other electrochemical parameters namely protection efficiency (*P_eff_*), polarization resistance (*R_p_*), and electrochemical porosity (*EP*). The protection efficiency (*P_eff_* ) was calculated using the Equation (1).

(1) $$P_{eff}=100\left(1-\frac{j_{corr}}{j_{corr}{}^{0}}\right)$$

where *j_corr_* and *j_corr_*^0^ indicate the corrosion current densities in the presence and absence of the coating, respectively [37,38]. The polarization resistance (*R_p_*) can be calculated using the Stern-Geary equation [18].

(2) $$R_{p}=\frac{b_{a}b_{c}}{2.3\;i_{corr}\;\left(b_{a}+b_{c}\right)}$$

where *b_a_* and *b_c_* are the slopes of the anodic and cathodic branches of the Tafel plot.

Finally, the electrochemical porosity (*EP*) of the samples (ii-iv) was calculated using Equation (3) [18].

(3) $$EP=\left(\frac{R_{pcontrol}}{R_{pcoated}}\right)\times 10^{-\left(\frac{\Delta V_{corr}}{b_{a}}\right)}\times 100$$

where *R_pcontrol_* and *R_pcoated_* are the polarization resistances of the control and coated samples, respectively, and ∆*V_corr_* is the difference in the corrosion potentials inferred from the polarization curve.

## 3. Results and Discussion

### 3.1. Material Characterization and Potentiodynamic Polarization

The measured film thicknesses were about 97 ± 4 nm for TiO_2_ and 105 ± 3 nm for Al_2_O_3_ on Al substrate. The thickness of the TiO_2_ film is in agreement with previous work of Chiappim et al. that deposited by thermal ALD TiO_2_ thin films on Si(100) surface at 100 °C using TiCl_4_ + H_2_O precursors [29]. For Al_2_O_3_ thin film on Al substrate, the measured thickness of 105 ± 3 nm was slightly higher than reported in literature, i.e. around 100 nm [39]. It is important to highlight that, although not focused here, our earlier studies on thickness dependence with number of cycles demonstrate that the process at 100 °C behaves as a real ALD [33].

The structural properties of TiO_2_ and Al_2_O_3_-coated aluminum were characterized by GIXRD (spectra not show here). The peaks observed at angle 2θ = 44.73°, 65.13° and 78.23° can be assigned to the diffraction from aluminum (JCPDS#04-0787), while no peaks coming from the TiO_2_ and Al_2_O_3_ films were detected, which suggests that both materials are amorphous. In fact, as observed by Chiappim et al. for TiO_2_ films [29] and by Miikkulainen et al. for Al_2_O_3_ films [40], ALD performed at temperature about 100 °C is not capable to initiate the crystallization process during the film growth.

**Figure 1:**
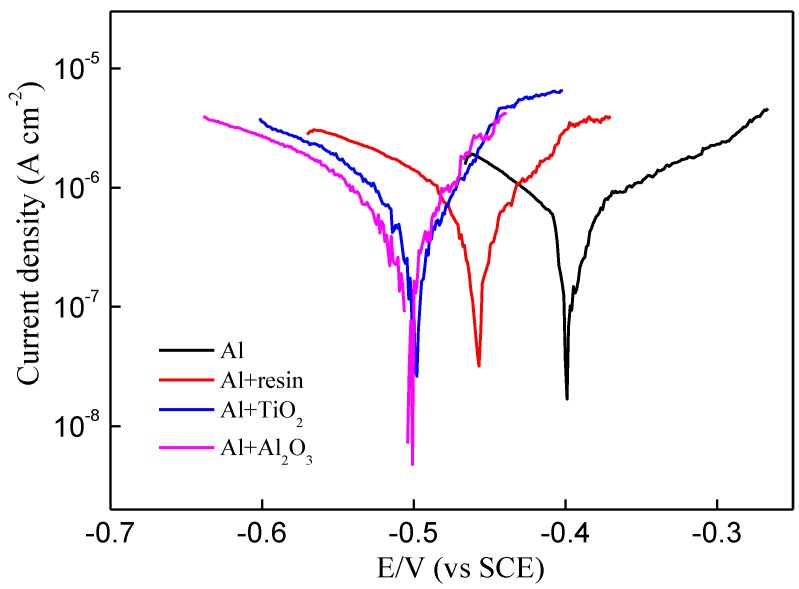
Polarization curves of the studied samples.

After the first considerations about the TiO_2_ and Al_2_O_3_ thin films, the next analysis concerns the corrosion assays in beer electrolyte. LSV tests were used to investigate the corrosion resistance of the coatings. The electrochemical parameters obtained from the polarization curves (Figure 1) using Tafel plot and equations (1–3) are summarized in Table 1. The smooth shape of the polarization curves of the samples, with only a single peak at the corrosion potential, indicates that the film is electrochemically inert [18]. For bare Al, the corrosion potential (*V_corr_*) is about −0.40 V, while the value is −0.46 V for the commercial resin-coated Al, −0.50 V for TiO_2_-coated Al, and −0.55 V for Al_2_O_3_-coated Al sample. The shift of the *V_corr_* to more negative potential indicates an improved corrosion resistance. The ALD coatings led a considerable increase in protection efficiency (*P_eff_*), i.e. 86.3% for TiO_2_-coated Al and 80% for Al_2_O_3_-coated Al. Note here that the *P_eff_* of the resin-coated Al is only 7.1%. Concerning the electrochemical porosity (*EP*), a direct correlation with *P_eff_* can be observed from Table 1, where TiO_2_-coated Al had the lowest value (11.4%). In comparison with the work of Daubert et al. [18], where TiO_2_ with *EP* of 0.9% and Al_2_O_3_ with *EP* of 0.1% were obtained by ALD at 150 °C on copper substrate, our results indicate the deposition of films on aluminum with higher *EP*. One possible explanation is due to lower ALD process temperature of our work (100 °C)—it is known that at low process temperature the ALD films tend to have lower densities [41]; however, more studies are needed to better explain this difference between *EP* values.

**Figure 2:**
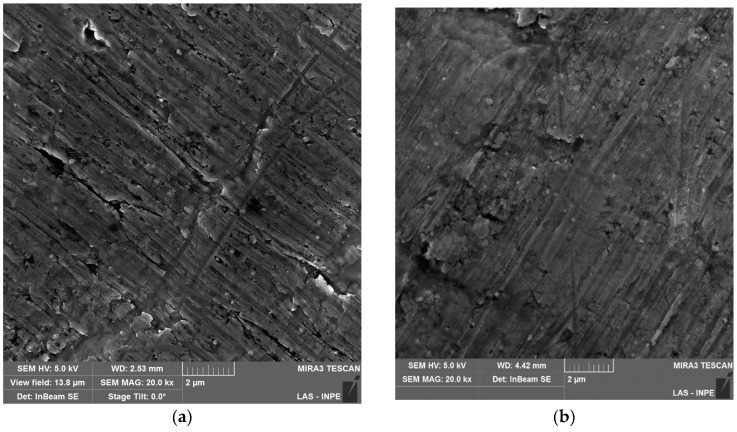

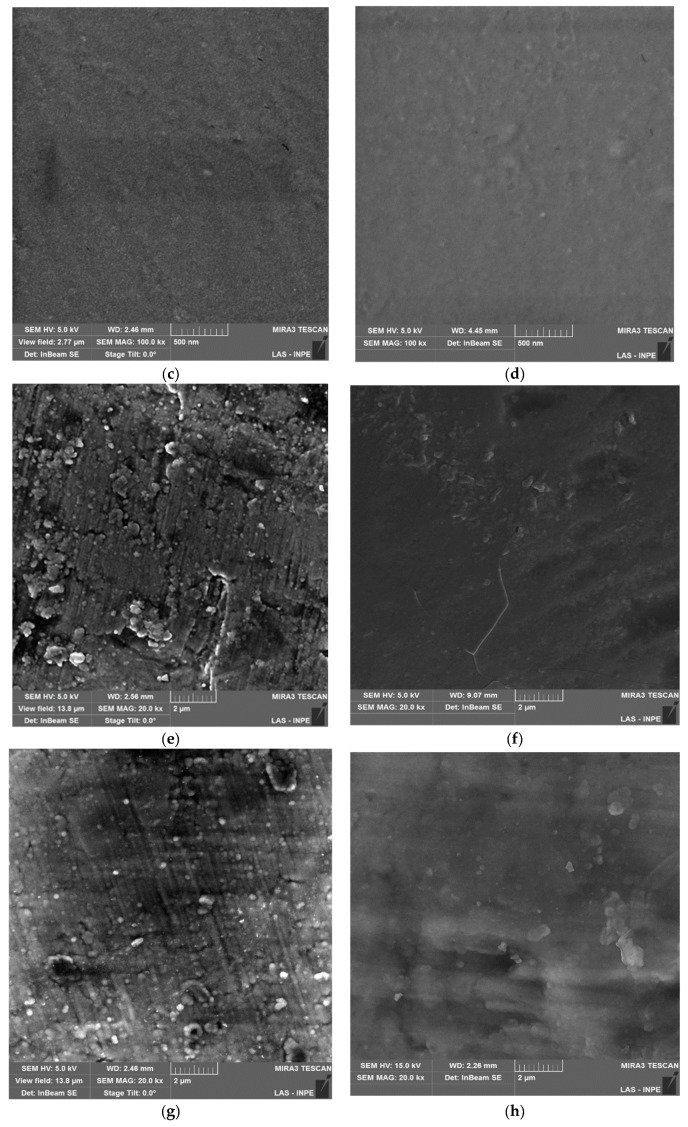
Scanning electron microscopy (SEM) micrographs of the studied samples: (**a**) bare Al (**b**) commercial resin-coated Al, (**c**) TiO_2_-coated Al, (**d**) Al_2_O_3_-coated Al, in the condition of as-deposited; (**e**) bare Al, (**f**) commercial resin-coated Al, (**g**) TiO_2_-coated Al, (**h**) Al_2_O_3_-coated Al in the condition after corrosion process. Here, the corrosion time of the samples was of 10 min.

Figure 2 shows the SEM micrographs of the pristine and corroded samples. The micrograph analyzes corroborate with the results obtained from the polarization curves. For the bare Al sample, a rough surface is observed where the grooves resulting from the rolling process of aluminum cans (Figure 2a). By other hand, for resin-coated Al, it can be observed a smooth surface, but with presence of pores (Figure 2c). After the corrosion period of 10 min, both bare and resin-coated Al maintained the initial morphology (Figure 2b,d). The formation of the TiO_2_ layer promoted a slight modification of the Al surface morphology with formation of some grains along the surface (Figure 2e). Indeed, thin amorphous TiO_2_ films tend to follow the surface roughness of substrate being coated [29]. Although the as-deposited surface appears rough, the 97 nm TiO_2_ coating promoted a reduction in *EP* by almost 90% compared with bare Al and a resulting *P_eff_* of 86.3%. This decreasing of *EP* can be result of corrosion process, changing the morphology of TiO_2_ film surface (Figure 2f). It is important to highlight that the TiO_2_ film thickness oscillated within the standard deviation after corrosion process. As with TiO_2_-coated Al, the alumina-coated sample reflects the substrate morphology with grain formation along the surface (Figure 2g). The 105 nm Al_2_O_3_ coating promoted a decreasing of *EP* of around 80% and a resulting *P_eff_* of 80%. The LSV results also corroborates with SEM micrographs for Al_2_O_3_-coated Al that shows a smoother surface with no considerable modification in the initial film thickness after corrosion process (Figure 2h). Although inferior to the TiO_2_ result, the corrosive protection of the alumina-coated sample was superior to that of the resin-coated sample, which was only 7.1%.

**Figure 3:**
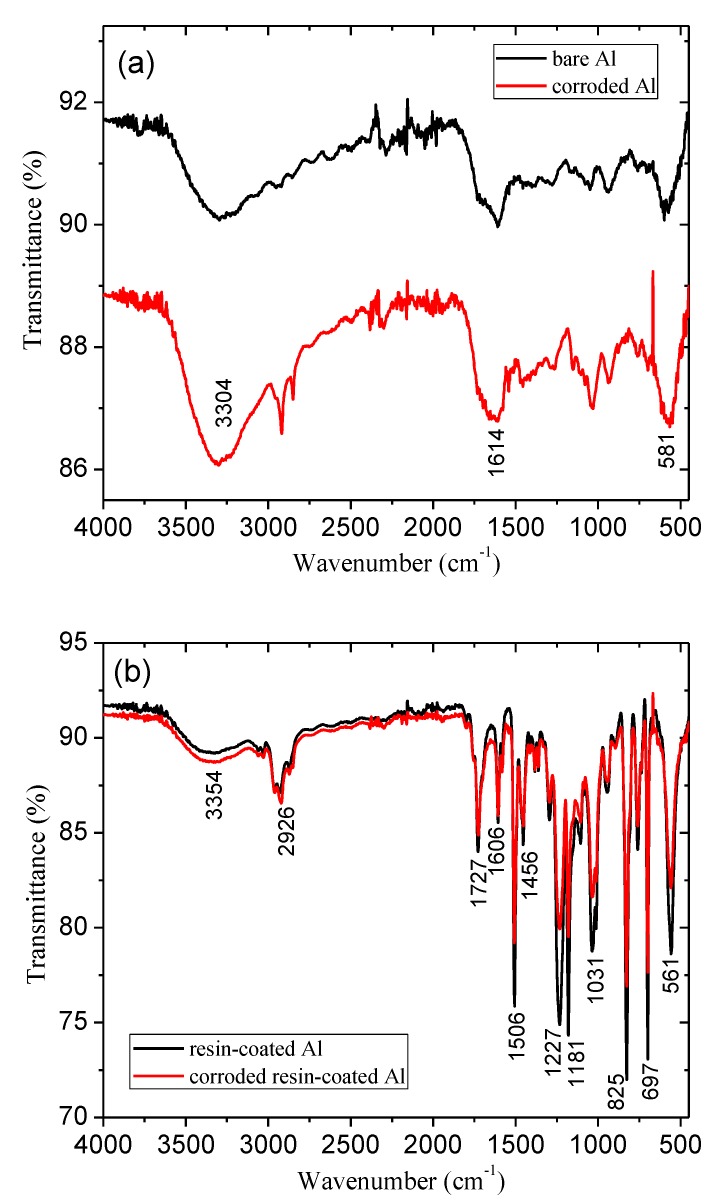

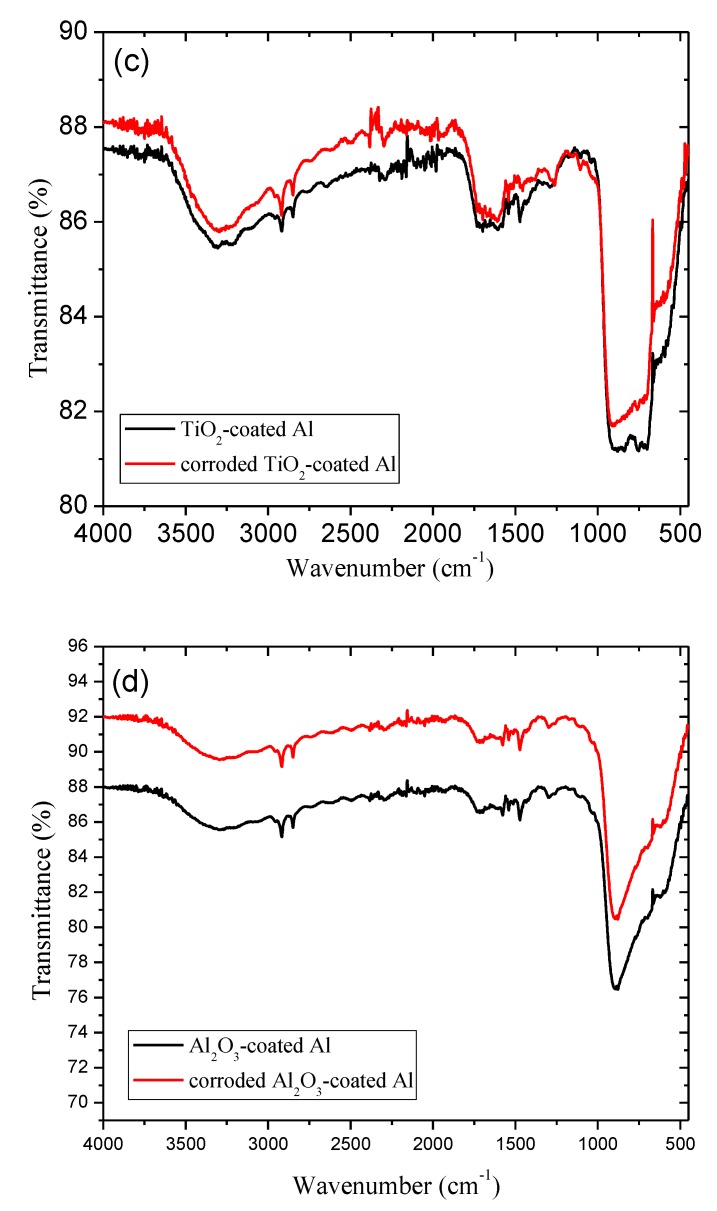
FT-IR spectra of the studied samples in the condition of as-deposited and after corrosion process. (**a**) control or bare Al, (**b**) commercial resin-coated Al, (**c**) TiO_2_-coated Al, (**d**) Al_2_O_3_-coated Al.

To complement the LSV and SEM results, FT-IR measurements were performed in pristine and corroded samples. The resulting spectra are shown in Figure 3. Initially, the analysis of the pure aluminum substrate was performed before and after the corrosion process in beer electrolyte (Figure 3a). The spectra show a wide O–H stretch band in the range of 3000 to 3700 cm^−1^ and in the band centered at 1614 cm^−1^. The strong and wide absorption band, centered at 581 cm^−1^, probably results from the Al–O stretching vibrations and the Al–OH vibrational mode [42]. Based on the spectra of Figure 3a, it is possible to observe that the aluminum after undergoing the process of corrosion in the beer media, increased the adsorption of water species, as well as formed a superficial oxide. However, aluminum is not protected by an adherent oxide layer at low pH because the superficial aluminum oxide is not stable. Hence, if the coating is not a perfect barrier, and low pH solution is able to reach the underlying aluminum, then it should fail rapidly from corrosion [8].

The spectra shown in Figure 3b are relative to the aluminum substrate coated with epoxy resin. The specific elongation vibration band for the C–O of the epoxy ring is observed at 825 cm^−1^. Bands formed between 1587 and 1668 cm^−1^ are assigned to the asymmetric C–O and C=O bonds, respectively. The C–O–C stretch is observed at 1031 cm^−1^, relative to the reaction of hydroxyl groups with epoxy ring. A small characteristic band at O–H appeared at 3300–3500 cm^−1^, relative to the reaction of the epoxy with the amine to form hydroxyl groups [43]. Note that the intensity of the peaks relative to the epoxy decreased after the corrosion process in beer electrolyte, indicating a possible reduction in the amount of material. However, as the pH of the medium changed from pH = 4.12 ± 0.02 to pH = 4.17 ± 0.03 after the immersion time of 1h10min (OCP + corrosion time), this amount of material may be very lower, even for bare Al substrate.

With the coating of the aluminum substrate with TiO_2_ film (Figure 3c), a significant change in the bands between 500–1000 cm^−1^ compared to the bare-Al sample could be observed (Figure 3a). These bands are probably related to Ti–O bonds. Note that there was a small variation in the intensity of the O–H bonds after the corrosion process, indicating good chemical stability of the TiO_2_ film. This result is confirmed by the LSV data. In relation to aluminum sample coated with alumina (Figure 3d), a behavior very similar to TiO_2_ was observed.

### 3.2. Electrochemical Impedance Spectroscopy

**Figure 4:**
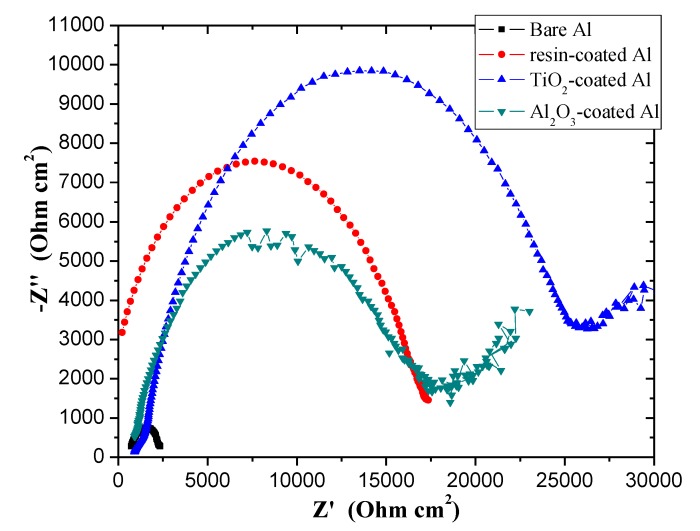
Nyquist plots for the studied samples.

**Figure 5:**
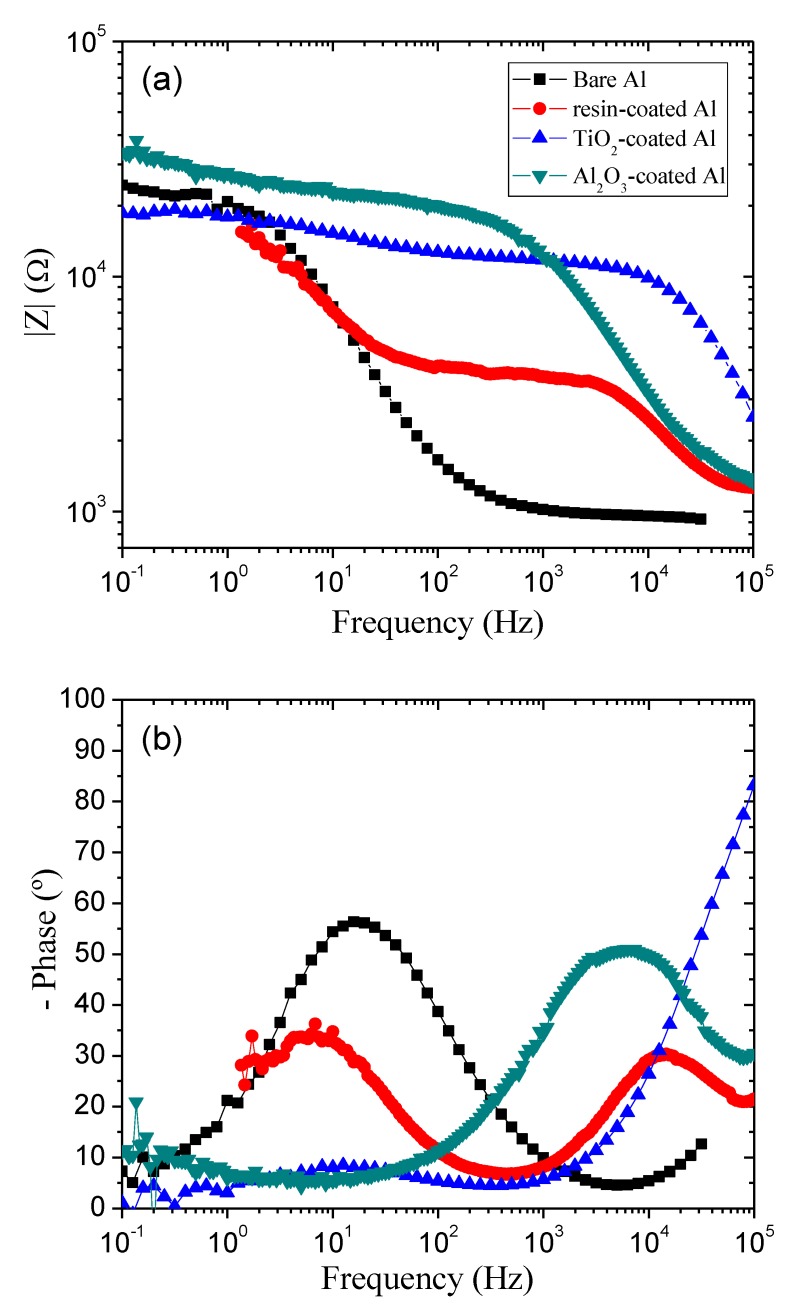
Bode magnitude (**a**) and Bode phase (**b**) plots of the electrochemical impedance spectroscopy for the studied samples.

The EIS allows a detailed understanding of the electrical characteristics of the electrode–solution interface, by means of the application of a small alternating voltage disturbance in the electrochemical system and recording from which the impedance is calculated [44]. Figure 4 and Figure 5 present the complex plane (Nyquist) plot and the Bode plots of the samples, respectively. Examination of Figure 4 shows that all films are very effective in terms of increasing impedance of the Al substrate. The samples show capacitive arc characteristic that indicates corrosion protection, although some samples also present resistive characteristic in the region of high frequencies, which is attributed to non-ideality of the coatings in complete blocking the corroding species (such as water, oxygen, and ions) [36]. Concern the capacitive arc diameter, the resin-coated sample increased by approximately 11 times the diameter in comparison with bare Al, indicating the ability of the resin to protect the aluminum in contact with the beer electrolyte. When compared with uncoated aluminum, the TiO_2_ film provided an increase by a factor of 14 the arc diameter. Instead, the Al_2_O_3_ film provided an increase of the arc diameter by 12.

For a better analysis of the EIS data, corresponding Bode magnitude and Bode phase plots are presented in Figure 5a,b, respectively. Bode magnitude plot represents the ratio of the amplitude of alternating voltage to alternating current versus frequency, irrespective of their phase shift. On the other hand, the Bode phase plot represents the phase difference between alternating voltage and alternating current versus frequency, irrespective of their amplitudes [36]. Both plots contain information which is not directly extractable from Nyquist plots. Figure 5a shows an increase in impedance magnitude for Al_2_O_3_-covered sample. However, the plots for all samples approach to each other at the lowest frequency, reaching values between 20–35 kΩ. s. Bode phase plots in Figure 5b are additional feature of EIS data. A characteristic of Bode phase plot is its ability to identify the predominant electrical behavior of the system in a given frequency range. For example, capacitive, resistive and mixed capacitive-resistive behavior appear as −90, 0, and 0 to −90 degrees lines, respectively [36]. Bode phase plots in Figure 5b shows completely different behavior for samples coated and uncoated. The first knee observed for covered samples, and more evident for commercial resin-coated Al, corresponds to the capacitive-resistive transition of the Bode phase plots [36]. In addition, the resin-coated Al plot resembles to two-time constants with same order of magnitude. These two processes are ascribable to coating and electrolyte–Al interface. The low resistance and high capacitance arise from high porosity of the coating [36], corroborating with LSV results. The hase angle of −50 degrees in high frequency for Al_2_O_3_-covered Al is characteristic of capacitor. Instead, phase angle of −85 degrees in high frequency for TiO_2_-covered Al is characteristic of a pure capacitor acting as efficient blocking coating. The lower efficiency of the Al_2_O_3_ film in comparison with TiO_2_ is intriguing and deserves further investigations.

For extraction of more information from EIS data, the data were fitted to an electrical equivalent circuit. A two-time constant circuit was adopted for this purpose [36,45]. However, due to system heterogeneities, the coincidence of experimental and fitted values was poor and therefore, the capacitor elements were replaced by constant phase elements (CPE). In the equivalent circuit, *R_s_* is the solution/electrolyte resistance and *R_p_* is the polarization resistance. The values of CPE, *R_s_* and *R_p_* are presented in Table 2. As can be seen, *R_p_* increased considerably when aluminum was coated, especially with the ALD films investigated here. As *R_p_* is directly related to the coating resistance, again the higher efficiency of the TiO_2_ film compared to the Al_2_O_3_ films is evident. The CPE and *R_s_* parameters are also optimized for TiO_2_.

## 4. Conclusions

In conclusion, TiO_2_ and Al_2_O_3_ thin films have been coated onto Al substrates using ALD method in order to improve the corrosion resistance for food packaging application. Here, we evaluated for the first time the initial corrosion barrier resistance of these ALD films in commercial beer electrolyte and performed a comparison with commercial resin-coated Al. GIXRD, SEM and profilometry analyzes showed that the films have an amorphous structure, a morphology that follows Al substrate surface, and a thickness of around 100 nm. Additionally, it was possible to see from SEM micrographs that the films entirely shield the substrates. The electrochemical measurements show that the equilibrium corrosion potential shifts from −0.40 V for bare Al to −0.46 V for the commercial resin-coated Al, −0.50 V for TiO_2_-coated Al, and −0.55 V for Al_2_O_3_-coated Al. By means of a comparative evaluation of the samples, it was possible to visualize the behavior pattern of the capacitive arc and the corrosion process in beer electrolyte. From the analysis of LSV data was verified that ALD coatings promoted a considerable increase in corrosion barrier efficiency being 86.3% for TiO_2_-coated Al and 80% for Al_2_O_3_-coated Al in comparison with 7.1% of commercial resin-coated Al. This is mainly due to the lower electrochemical porosity—11.4% for TiO_2_-coated Al and 20.4% for Al_2_O_3_-coated Al, in comparison with 96% of the resin-coated Al, i.e. an increase of up to twofold in the protection of Al when coated with TiO_2_, when compared to aluminum coated. The impedance results confirm that TiO_2_-coated Al have good corrosion resistance in beer electrolyte. It was observed from Bode plots in high frequency that the phase angle of −50 degrees for Al_2_O_3_-covered Al is characteristic of capacitor, while the phase angle of −85 degrees for TiO_2_-covered Al is characteristic of a pure capacitor. This result shows the TiO_2_-covered Al acting as efficient blocking coating. Also, it was showed that the reduced corrosion barrier efficiency of the Al_2_O_3_ film in comparison with TiO_2_ for beer electrolyte once SEM and FT-IR analyses did not show drastic changes in the films after the corrosion assays. A further investigation is being carried out with standard NaCl electrolytes in order to clarify this low efficiency of Al_2_O_3_ film. The results of our initial analysis of the protective performance of ALD TiO_2_ and Al_2_O_3_ coatings demonstrated that they could be an alternative for the corrosion protection of aluminum food packaging. In future studies, we can also further explore their protective properties for this application by combining Al_2_O_3_ and TiO_2_ to produce Al_2_O_3_-TiO_2_ nanolaminates. Finally, to evaluate the shelf life time of beer or other carbonated beverage, whether in stock in the industry or in supermarket shelves, it is necessary to evaluate the effect of the immersion time over long periods (several hours or days).

## Figures and Tables

**Table 1:** Corrosion properties of the studied samples obtained from polarization curves.

Samples	*V_corr_* (V vs. Ag/AgCl)	*j_corr_* (10^−6^ A.cm^2^)	*R_p_* (10^4^ Ω/cm^2^)	EP (%)	*P_eff_* (%)
**Al**	−0.40	9.06	2.77	100.0	-
**Al + resin**	−0.46	7.56	2.20	96.0	7.1
**Al + TiO_2_**	−0.50	1.24	2.19	11.4	86.3
**Al + Al_2_O_3_**	−0.55	1.79	2.27	20.4	80.0

**Table 2:** Values of the elements of the equivalent electric circuit used to simulate the electrochemical impedance spectroscopy measurements of the samples in the beer electrolyte. CPE—constant phase element. *R_s_*—solution/electrolyte resistance. *R_p_*—polarization resistance. n—circuit fitting parameter.

Sample	CPE (F)	*R_s_* (Ω)	*R_p_* (Ω)	n (%)
Al	7.37 × 10^−9^	1056.3	2.841.8	99.8
Al + V	4.66 × 10^−9^	979.7	17.905.0	99.8
Al + TiO_2_	3.52 × 10^−7^	1682.8	32.619.0	99.7
Al + Al_2_O_3_	7.52 × 10^−9^	1220.2	27.442.0	99.6
